# Exercise training reverses cardiac aging phenotypes associated with heart failure with preserved ejection fraction in male mice

**DOI:** 10.1111/acel.13159

**Published:** 2020-05-22

**Authors:** Jason D. Roh, Nicholas Houstis, Andy Yu, Bliss Chang, Ashish Yeri, Haobo Li, Ryan Hobson, Carolin Lerchenmüller, Ana Vujic, Vinita Chaudhari, Federico Damilano, Colin Platt, Daniel Zlotoff, Richard T. Lee, Ravi Shah, Michael Jerosch‐Herold, Anthony Rosenzweig

**Affiliations:** ^1^ Corrigan Minehan Heart Center Massachusetts General Hospital Harvard Medical School Boston MA USA; ^2^ Department of Cardiology, Angiology, and Pulmonology University Hospital Heidelberg Heidelberg Germany; ^3^ Department of Stem Cell and Regenerative Biology Harvard Stem Cell Institute Harvard University Cambridge MA USA; ^4^ Department of Radiology Brigham and Women’s Hospital Harvard Medical School Boston MA USA

**Keywords:** aging, cardiac, cardiovascular, exercise, heart failure, RNA sequencing

## Abstract

Heart failure with preserved ejection fraction (HFpEF) is the most common type of HF in older adults. Although no pharmacological therapy has yet improved survival in HFpEF, exercise training (ExT) has emerged as the most effective intervention to improving functional outcomes in this age‐related disease. The molecular mechanisms by which ExT induces its beneficial effects in HFpEF, however, remain largely unknown. Given the strong association between aging and HFpEF, we hypothesized that ExT might reverse cardiac aging phenotypes that contribute to HFpEF pathophysiology and additionally provide a platform for novel mechanistic and therapeutic discovery. Here, we show that aged (24–30 months) C57BL/6 male mice recapitulate many of the hallmark features of HFpEF, including preserved left ventricular ejection fraction, subclinical systolic dysfunction, diastolic dysfunction, impaired cardiac reserves, exercise intolerance, and pathologic cardiac hypertrophy. Similar to older humans, ExT in old mice improved exercise capacity, diastolic function, and contractile reserves, while reducing pulmonary congestion. Interestingly, RNAseq of explanted hearts showed that ExT did not significantly modulate biological pathways targeted by conventional HF medications. However, it reversed multiple age‐related pathways, including the global downregulation of cell cycle pathways seen in aged hearts, which was associated with increased capillary density, but no effects on cardiac mass or fibrosis. Taken together, these data demonstrate that the aged C57BL/6 male mouse is a valuable model for studying the role of aging biology in HFpEF pathophysiology, and provide a molecular framework for how ExT potentially reverses cardiac aging phenotypes in HFpEF.

## Graphical abstract

Heart failure with preserved ejection fraction (HFpEF) is the most common type of heart failure in older adults, but has no effective pharmacological therapies. This study shows that exercise training can reverse cardiac aging biology and phenotypes that contribute to HFpEF, and identifies cell cycle pathways as potential novel therapeutic targets for age‐related HFpEF.

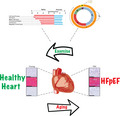

## 1 INTRODUCTION

Heart failure with preserved ejection fraction (HFpEF) is a complex, heterogenous clinical syndrome strongly associated with advanced age (Upadhya, Taffet, Cheng, & Kitzman, 2015). It now represents the most common form of HF in older adults with a growing prevalence largely attributed to global population aging (Dunlay, Roger, & Redfield, 2017). Unfortunately, prognosis for older adults with HFpEF remains poor. HF remains the leading cause of hospitalization amongst persons over 65 years old, and nearly 1/3 of all older adults hospitalized for HF are either readmitted or dead within 90 days of discharge (Upadhya et al., 2015). Notably, no pharmacological agent, including neurohormonal antagonists and nitrate derivatives (Borlaug et al., 2018; Massie et al., 2008; Pitt et al., 2014; Redfield et al., 2015), has improved survival in HFpEF, making it one of the largest unmet needs in geriatric and cardiovascular medicine (Parikh et al., 2018).

The reasons we lack effective pharmacological interventions in HFpEF are multifold, but largely stem from an incomplete understanding of the underlying mechanisms that drive HFpEF pathophysiology (Roh, Houstis, & Rosenzweig, 2017). In older adults, molecular, structural, and functional changes associated with cardiac aging have long been hypothesized to be major contributors to HFpEF (Roh, Rhee, Chaudhari, & Rosenzweig, 2016; Strait & Lakatta, 2013; Upadhya et al., 2015). However, whether the biology of cardiac aging can be targeted for HFpEF therapy is unclear.

Despite limited success of current pharmacological agents, aerobic exercise training (ExT) has emerged as one of the most effective strategies for improving functional outcomes in older adults with HFpEF (Edelmann et al., 2011; Kitzman et al., 2016; Kitzman, Brubaker, Morgan, Stewart, & Little, 2010; O’Connor et al., 2009). Whether ExT can alter cardiac aging phenotypes that contribute to HFpEF pathophysiology, however, is controversial. While some studies have suggested that ExT improves diastolic function and cardiac reserves in older HFpEF patients, others have shown that ExT has minimal effects on these cardiac aging phenotypes (Angadi et al., 2015; Edelmann et al., 2011; Haykowsky et al., 2012; Kitzman et al., 2010, 2016; Nolte et al., 2014). Moreover, the molecular mechanisms by which ExT potentially improves cardiac performance in HFpEF are unknown.

This study addresses these critical issues by first demonstrating that the aged C57BL/6 male mouse is particularly well suited for studying the role of aging biology in HFpEF pathophysiology. Using this age‐related HFpEF model, we then show that ExT partially reverses many, but not all, of the cardiac aging phenotypes associated with HFpEF. Finally, combining RNAseq profiling and ExT in an integrated platform for therapeutic target discovery provides a scientific rationale for why previous drug targets may have failed in clinical HFpEF trials and implicates alternative biological pathways as candidates for therapeutic intervention in age‐related HFpEF.

## 2 RESULTS

### 2.1 Cardiac functional HFpEF phenotyping

Since advanced age represents one of the dominant risk factors for HFpEF, we hypothesized that old mice might share hallmark phenotypes present in human HFpEF. To evaluate the aged mouse as a HFpEF model, we first set criteria based on the most common pathophysiologic features seen in clinical HFpEF (Borlaug, 2014; Mohammed et al., 2015), which included the following: (a) preserved left ventricular (LV) systolic function, measured by ejection fraction (EF) or fractional shortening (FS); (b) impaired exercise capacity; (c) impaired contractile or chronotropic reserves; (d) increased intracardiac filling pressures, B‐type natriuretic peptide (BNP) expression, or pulmonary congestion; and (e) histologic features consistent with pathologic cardiac hypertrophy. Using these prespecified criteria, we performed comprehensive phenotyping in young (3–4 months), old (24–26 months), and very old (28–30 months) C57BL/6 male mice. Two stages of advanced age were used to determine whether HFpEF phenotypes progressed in the late stages of the murine lifespan to further investigate the role of aging biology in HFpEF pathophysiology.

**FIGURE 1:**
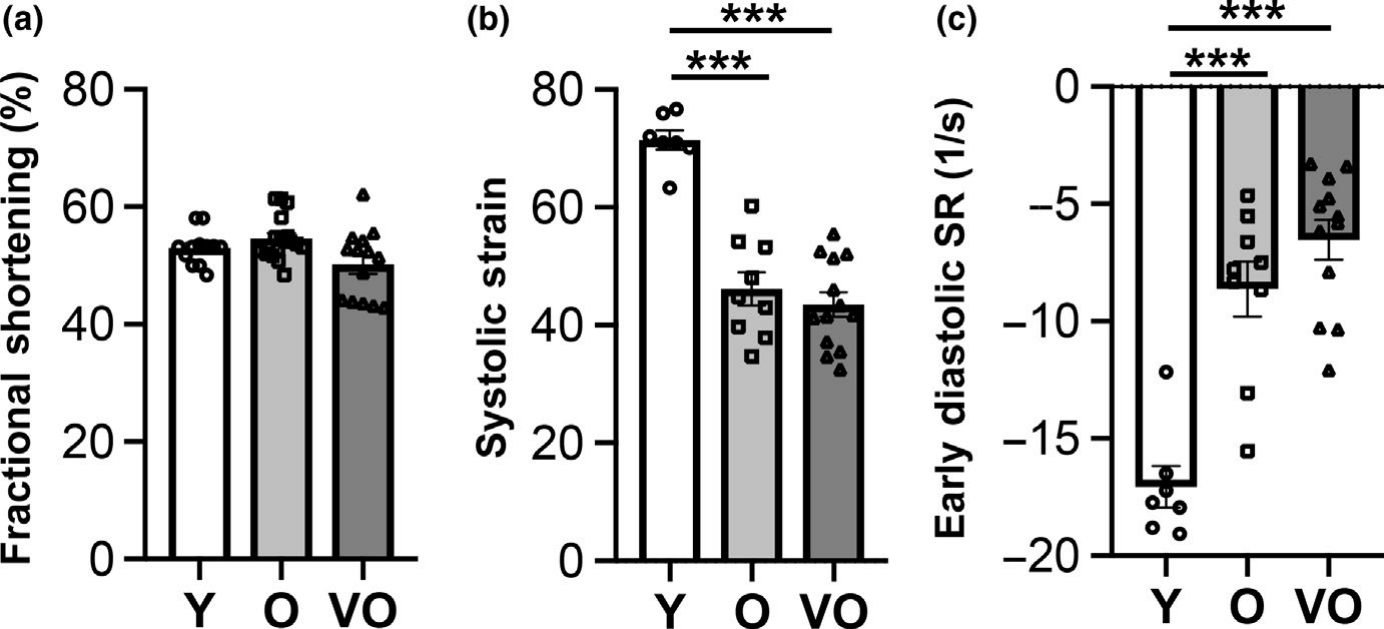
Age‐related changes in resting cardiac function in C57BL/6 male mice are similar to cardiac functional phenotypes in human HFpEF. Nonsedated transthoracic echocardiography in C57BL/6 male mice at 3–4 months (young (Y), *n* = 12), 24–26 months (old (O), *n* = 17), and 28–30 months (very old (VO), *n* = 14). (a) Fractional shortening, (b) systolic strain, (c) early diastolic strain rate (SR). Data shown as mean ± *SEM*, with all individual data points plotted. One‐way ANOVA with post hoc Tukey's test used for analyses. *p < .05, **p < .01, ***p < .001

Resting cardiac functional phenotypes were assessed using an extensive multimodality approach. Transthoracic echocardiography performed in a large cohort of animals (*n* = 43) found that LV fractional shortening was generally preserved in old and very old mice, compared to young mice (Figure 1a). Importantly, this defining feature of HFpEF was further validated in smaller subgroups using cardiac magnetic resonance imaging (Figure S1) and invasive intracardiac hemodynamic testing (Figure S2). Similar to human HFpEF, both old and very old mice displayed evidence of subclinical LV systolic dysfunction as reflected in reduced systolic strain (Figure 1b). Additionally, impaired myocardial relaxation, a marker of diastolic dysfunction commonly seen in HFpEF, was seen in aged mice (Figure 1c, Figure S2).

### 2.2 Exercise HFpEF phenotyping

**FIGURE 2:**
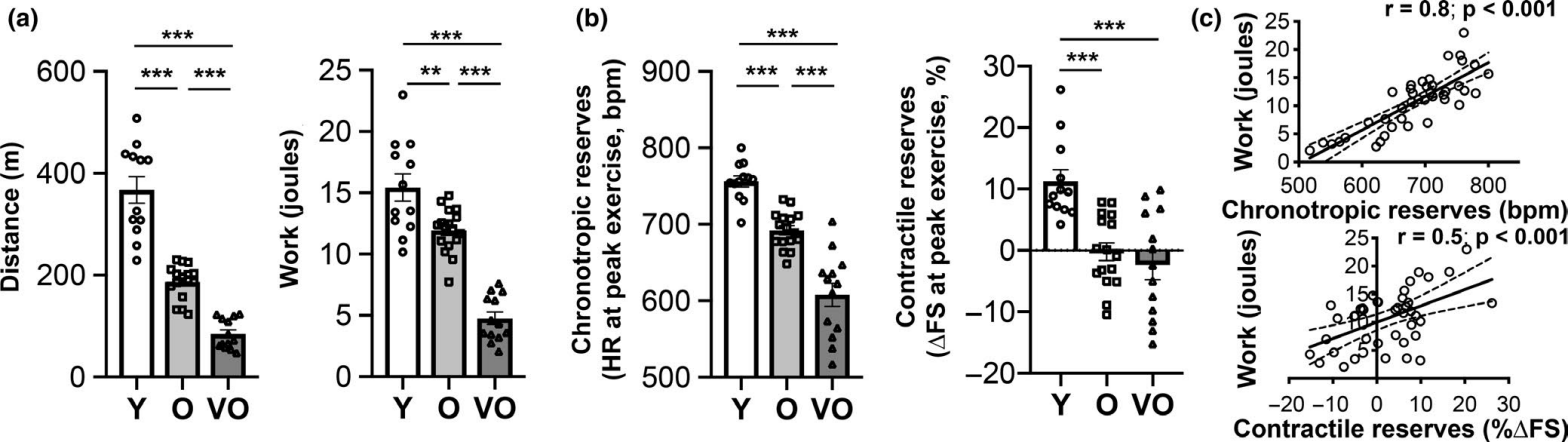
Progressive age‐related decline in exercise capacity and cardiac reserves in C57BL/6 male mice recapitulates exercise intolerance phenotypes in human HFpEF. Stress echocardiography testing in C57BL/6 male mice at 3–4 months (Y, *n* = 12), 24–26 months (O, *n* = 17), and 28–30 months (VO, *n* = 13). (a) Exercise capacity measured by total distance run and total work achieved (adjusted for body weight). (b) Chronotropic and contractile reserves measured at peak exercise. (c) Pearson correlation of exercise capacity (work) with chronotropic or contractile reserves. In panels a and b, data shown as mean ± *SEM*, with all individual data points plotted, and one‐way ANOVA with post hoc Tukey's test used for analyses. *p < .05, **p < .01, ***p < .001

The most consistent functional impairment seen in clinical HFpEF is exercise intolerance (Borlaug, 2014). Using stress echocardiography, we found that exercise capacity was markedly reduced in old and very old mice, even when adjusted for body weight (Figure 2a, Figure S3). Similar to human aging and HFpEF (Del Buono et al., 2019; Fleg et al., 2005; Strait & Lakatta, 2013), there was an age‐associated decline in exercise capacity that further progressed from 24 to 30 months (Figure 2a). Both chronotropic and contractile cardiac reserves decreased with age, although only chronotropic reserves continued to decline from 24 to 30 months (Figure 2b). Both chronotropic and contractile reserves correlated with exercise capacity (Figure 2c), suggesting that the impairments in cardiac reserves seen in older mice likely contribute to their age‐related decline in exercise capacity. Notably, these data are not only the first to demonstrate a marked age‐related decrement in cardiac reserves in C57BL/6 mice in the context of exercise, but also support the use of this model for studying the pathophysiology of age‐related HFpEF.

### 2.3 Histopathologic cardiac HFpEF phenotyping

**FIGURE 3:**
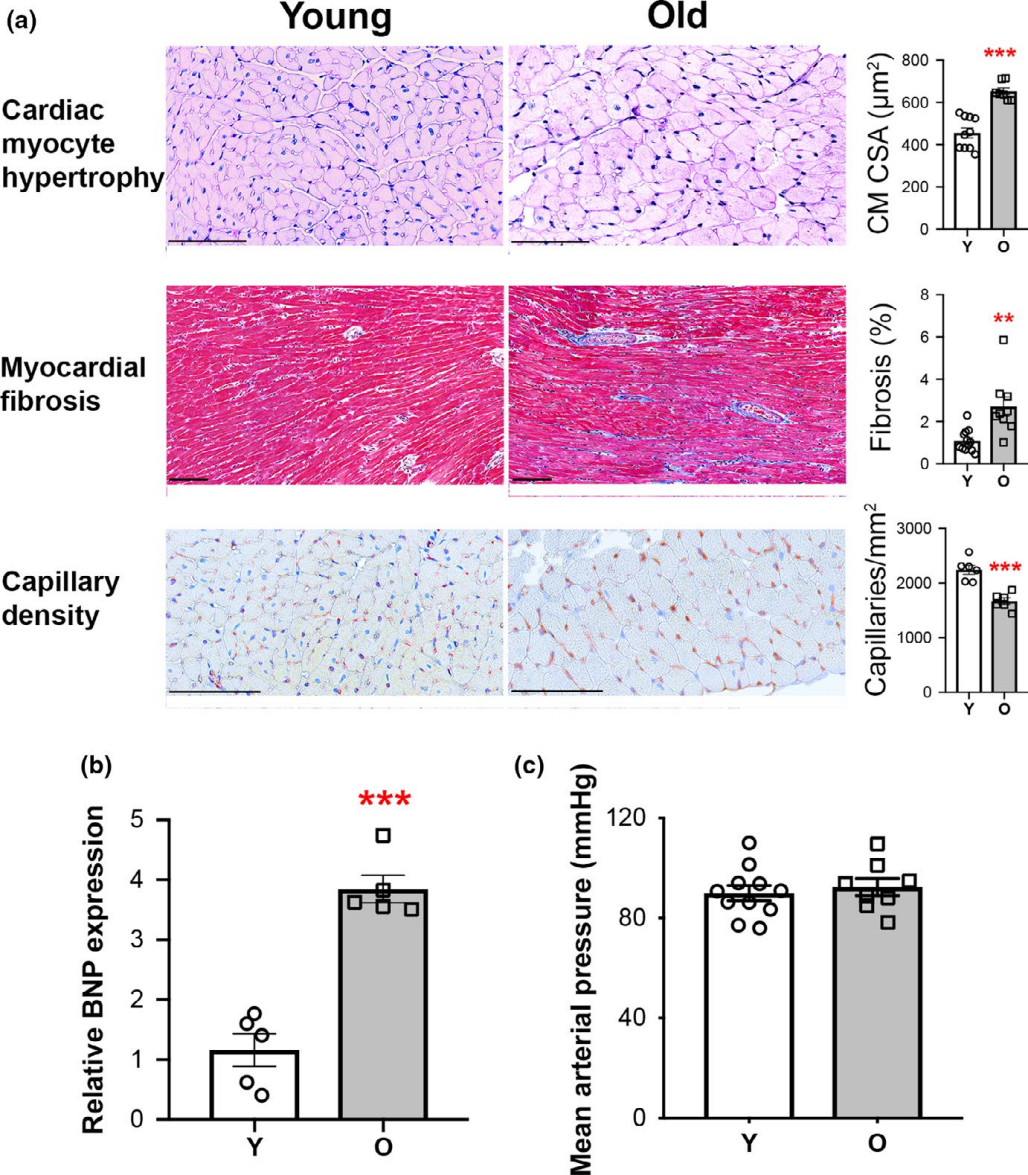
Pathologic cardiac hypertrophy in aged C57BL/6 male mice recapitulates histopathologic cardiac phenotypes in human HFpEF. (a) Cardiac histopathologic assessments of 4‐month (Y, *n* = 6–12) and 25‐ to 26‐month (O, *n* = 6–9) mice, including representative photomicrographs and quantification of cardiomyocyte cross‐sectional area (CM CSA), myocardial fibrosis, and capillary density. Scale bar = 100 µm. (b) Relative cardiac mRNA expression of BNP. *n* = 5/group. (c) Systemic mean arterial pressure (MAP) in Y (*n* = 11) versus O (*n* = 8) mice. For all panels, data shown as mean ± *SEM*, with all individual data points plotted. Unpaired Student's *t* test used for analyses. *p < .05, **p < .01, ***p < .001

In addition to the cardiac functional impairments and exercise intolerance seen in HFpEF, postmortem histopathologic characterization of hearts from HFpEF patients has revealed a common pathologic cardiac hypertrophy phenotype that includes increased cardiomyocyte size, fibrosis, and microvascular rarefaction (Mohammed et al., 2015). To determine whether aged C57BL/6 male mice exhibit these histopathologic HFpEF phenotypes, we first performed gravimetric analyses on young, old, and very old mice. Indexed lung weights, an indicator of pulmonary congestion, increased with age (Figure S4), suggesting that as these mice age, a progressive HF syndrome occurs that parallels the age‐related decline in functional exercise capacity (Figure 2). Although a significant increase in cardiac mass was seen between 4 and 26 months, there was no further increase after 26 months (Figure S4). Thus, we focused subsequent analyses on the young and old age groups. Old mice fully recapitulated the pathologic cardiac hypertrophy seen in human HFpEF, demonstrating increased cardiomyocyte size, myocardial fibrosis, and microvascular rarefaction (Figure 3a), which was associated with increased cardiac BNP expression, a biomarker of increased myocardial stress and HF (Figure 3b). Notably, blood pressures were similar between young and old mice (Figure 3c), suggesting that the pathologic cardiac remodeling seen in aged mice is not driven by overt hypertension but more likely related to processes intrinsic to cardiovascular aging.

### 2.4 Reversal of HFpEF phenotypes in aged C57BL/6 male mice with exercise training

**FIGURE 4:**
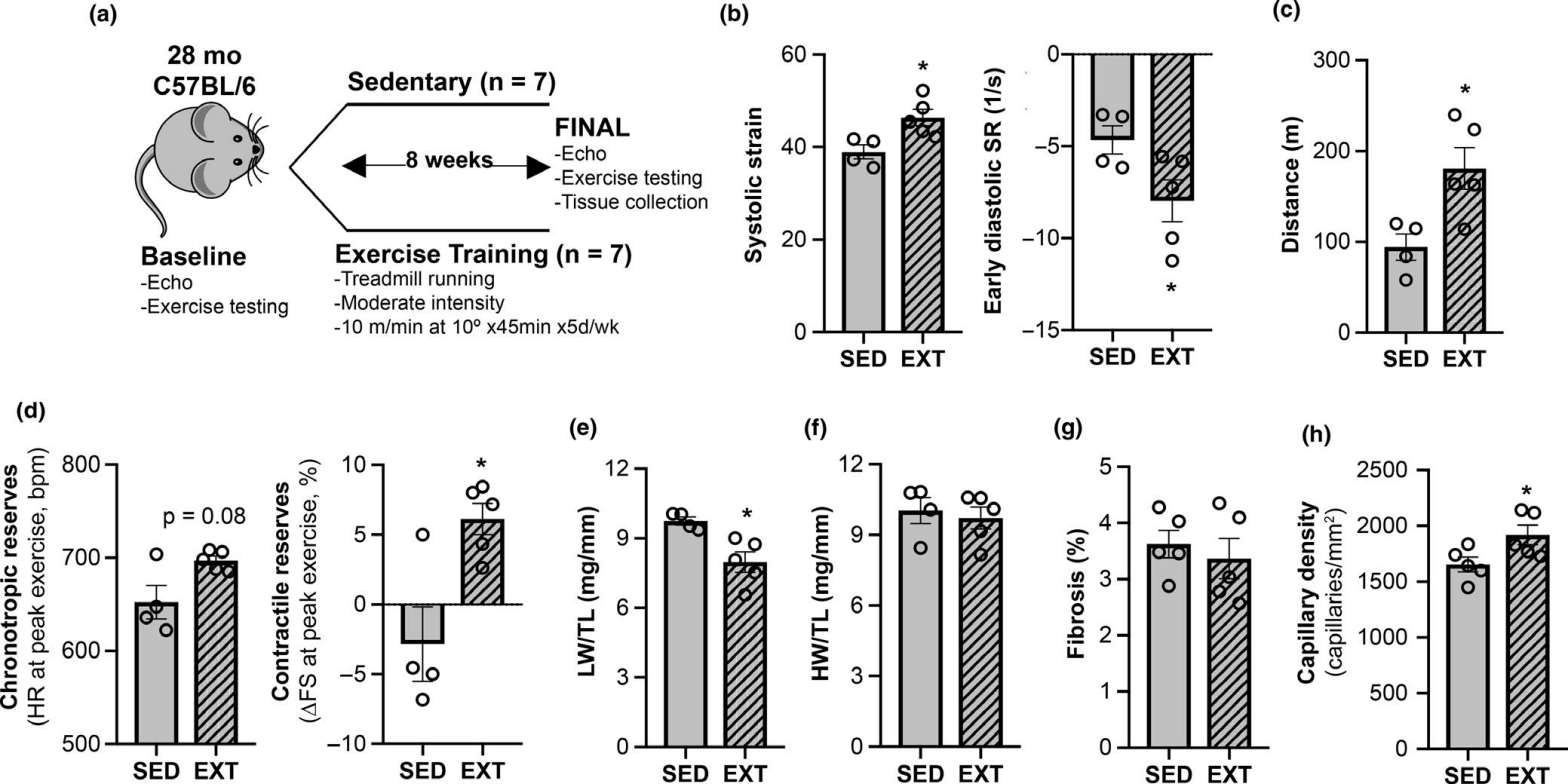
Aerobic exercise training reverses some HFpEF phenotypes in aged male mice. (a) Experimental design of exercise training substudy. Prior to study completion, 3 mice died in the sedentary group (SED) and 2 mice died in the exercise‐trained group (ExT). (b) Resting cardiac function by strain echocardiography. (c) Exercise capacity. (d) Chronotropic and contractile cardiac reserves. (e) Lung weight (LW) normalized to tibial length (TL). (f) Heart weight (HW) normalized to TL. (g) % Fibrosis. (h) Capillary density. For fibrosis and capillary density measurements, histologic sections from a SED animal that died one day prior to the final timepoint functional testing were included in the final analyses. Data shown as mean ± *SEM*, with all individual data points plotted. Unpaired Student's *t* test used for analyses. *p < .05, **p < .01, ***p < .001

Since aged C57BL/6 male mice recapitulate many of the HFpEF phenotypes observed in older humans, we next examined whether ExT could effectively reverse these age‐related HFpEF phenotypes. Mice were matched based on body weight and HFpEF phenotypes (Table 1) and then divided into either an 8‐week moderate‐intensity treadmill running protocol (45 min at 10 m/min at 10° incline) versus no intervention (normal sedentary lifestyle) (Figure 4a). ExT induced multiple functional improvements, some of which have been reported in older HFpEF patients (Angadi et al., 2015; Edelmann et al., 2011; Haykowsky et al., 2012; Kitzman et al., 2010, 2016; Nolte et al., 2014). Specifically, improvements in exercise capacity, systolic strain, diastolic function, contractile reserves, and pulmonary congestion were seen after eight weeks of ExT (Figure 4b–e, Figure S5). Although cardiac mass and fibrosis were not significantly changed, capillary density increased with ExT (Figure 4f–h).

**TABLE 1:** Baseline characteristics of 28‐month‐old C57BL/6 mice included in the exercise training substudy

Characteristic	Sedentary (*n* = 7)	Exercise (*n* = 7)	*p* value
Age (month)	28	28	n.a.
Sex	male	male	n.a.
Strain	C57BL/6	C57BL/6	n.a.
Weight (g)	31.6 ± 2.9	34.8 ± 3.5	.09
Resting cardiac function			
Fractional shortening (%)	52.9 ± 3.2	50.2 ± 2.8	.12
Exercise capacity			
Distance (m)	131.5 ± 26.3	136.2 ± 19.4	.71
Work (Joules)	7.0 ± 1.5	8.0 ± 0.7	.16
Lactate at peak exercise (mM)	8.2 ± 1.6	9.0 ± 1.1	.28
Cardiac reserves			
Chronotropic, HR at peak exercise (bpm)	665.5 ± 17.5	664.3 ± 46.8	.95
Contractile, Δ FS at peak exercise (%)	0.2 ± 8.4	2.2 ± 5.0	.59

Note

Prior to initiation of the exercise training protocol, no significant baseline differences in body weight, resting cardiac function, exercise capacity, or cardiac reserves were detected between the sedentary and exercise‐trained groups. *n* = 7/group. Data shown as mean ± *SEM*. Unpaired Student's *t* test used for analyses. *p* < .05 considered significant.

### 2.5 Exercise‐mediated transcriptome changes in the aged heart

**FIGURE 5:**
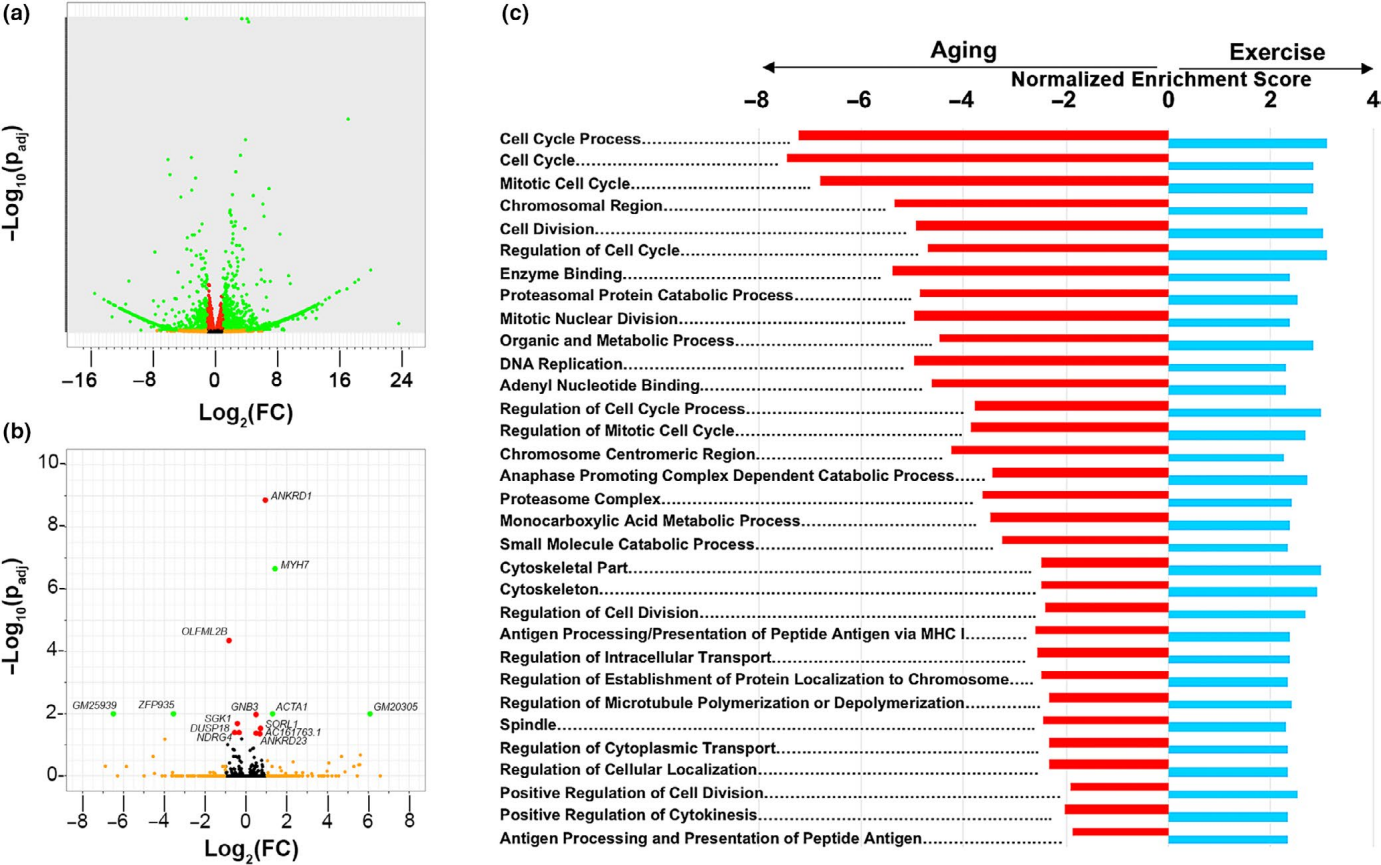
Reversal of cardiac aging pathways with exercise training. RNAseq analyses on cardiac tissue from young (4 months) sedentary, very old (30 months) sedentary, and very old (30 months) exercise‐trained (ExT) C57BL/6 male mice. ExT mice completed an 8‐week treadmill running protocol from 28 to 30 months prior to sacrifice. *n* = 3/group. (a) Volcano plot of differentially expressed genes between young and very old sedentary mice. (b) Volcano plot of differentially expressed genes between sedentary and ExT very old mice. (c) Most highly significant pathways that are differentially regulated in opposing directions by age (red, young sedentary vs. very old sedentary) and ExT (blue, very old sedentary vs. very old ExT). Only pathways with an FDR < 0.05 in both the aging and exercise‐trained cohorts with normalized enrichment scores (NES) in opposing directions are displayed. For volcano plots, black = nonsignificant; orange = log_2_(FC) ≥1; red = *p*_adj_ < .05; green = log_2_(FC) ≥1 + *p*_adj_ < .05

To explore the molecular pathways through which ExT modulates cardiac aging phenotypes associated with HFpEF, we performed RNAseq on a subset of cardiac samples from the ExT and sedentary aged mice. Fourteen genes (11 of which are known) were differentially expressed after adjusting for multiple hypothesis testing (Figure 5b, Table S1). Although RNAseq revealed only a small number of genes in the aged heart that were differentially regulated by ExT, gene set enrichment analysis did identify 479 significantly upregulated and 77 downregulated biological pathways (Tables S2 and S3). Interestingly, pathways associated with drug targets previously tested in clinical HFpEF trials, that is, adrenergic, renin–angiotensin–aldosterone (RAAS) and nitric oxide‐cGMP‐phosphodiesterase signaling pathways, were generally not significantly altered by ExT, although positive regulation of nitric oxide synthase biosynthesis reached our significance threshold (NES 1.67; FDR 0.24) and cellular responses to nitric oxide (NO) had a positive trend (NES 1.64, FDR 0.26) (Table 2). Rather the pathways most highly upregulated by ExT were predominantly cell cycle‐related processes, while the most highly downregulated were related to cellular respiration (Tables S2 and S3). Taken together, these data suggest that the cardiac benefits of ExT seen in older animals with HFpEF pathophysiology may be driven more by changes in these alternative biological pathways, as opposed to the neurohormonal pathways that are targeted with current HF medications.

**TABLE 2:** Effects of exercise training in aged hearts on biological pathways associated with previously tested drug targets in HFpEF

Pathway	NES	FDR
**Adrenergic system**		
Adrenergic receptor activity	0.89	0.81
Adrenergic receptor binding	0.69	0.94
Adrenergic receptor signaling pathway	0.88	0.82
Catecholamine binding	1.01	0.73
Catecholamine biosynthetic process	0.95	0.76
Catecholamine metabolic process	1.33	0.49
Catecholamine transport	1.18	0.60
Negative regulation of catecholamine secretion	1.01	0.73
Positive regulation of catecholamine metabolic process	−1.13	0.82
Regulation of norepinephrine secretion	−1.00	0.88
Response to epinephrine	−1.38	0.71
**Renin–angiotensin–aldosterone system**		
Angiotensin receptor binding	−0.85	0.93
Regulation of blood volume by renin–angiotensin	0.98	0.75
Regulation of systemic arterial blood pressure by circulating renin–angiotensin	1.48	0.38
Regulation of systemic arterial blood pressure by renin–angiotensin	1.45	0.40
Response to mineralocorticoid	−0.86	0.93
**Nitric oxide‐cGMP‐phosphodiesterase system**		
3’5’ cGMP phosphodiesterase activity	−1.56	0.57
Cellular response to nitric oxide	1.64	0.26
cGMP binding	−1.13	0.82
cGMP biosynthetic process	1.10	0.66
cGMP metabolic process	1.08	0.68
Negative regulation of nitric oxide metabolic process	0.95	0.76
Nitric oxide mediated signal transduction	−1.45	0.67
Nitric oxide metabolic process	1.29	0.52
Nitric oxide synthase binding	−1.08	0.84
Positive regulation of nitric oxide synthase activity	−0.96	0.90
Positive regulation of nitric oxide synthase biosynthetic process	1.67	0.24
Regulation of cGMP biosynthetic process	−1.03	0.86
Regulation of cGMP metabolic process	1.08	0.68
Regulation of nitric oxide biosynthetic process	−0.86	0.93
Regulation of nitric oxide synthase activity	−0.77	0.97
Regulation of nitric oxide synthase biosynthetic process	1.42	0.42
Response to nitric oxide	1.50	0.36

Note

Gene set enrichment analysis of RNAseq profiles from cardiac tissue of sedentary versus exercise‐trained aged male mice using the Gene Ontology pathway dataset. *N* = 3/group. NES = normalized enrichment score. False discovery rate (FDR) <0.25 considered significant.

### 2.6 Reversal of cardiac aging pathways by exercise training

To determine whether ExT potentially reverses cardiac aging biology associated with HFpEF, we next assessed how the cardiac transcriptome changes with normal aging to provide a comparison to the ExT‐induced transcriptome seen in the aged heart. Not surprisingly, RNAseq analyses identified a large number of genes that were differentially expressed in 30‐month‐old compared to 4‐month‐old hearts (Figure 5a, Table S4). Gene set enrichment analysis confirmed that biological processes implicated in cardiac aging were changing in the expected directions in our aged mice (Bergmann et al., 2009; Dai et al., 2009; Eisenberg et al., 2016; Hulsmans et al., 2018; Lee, Alison, Brand, Weindruch, & Prolla, 2002; Roh et al., 2016). Inflammation, cytokine production, complement activation, and extracellular matrix production pathways were upregulated in the aged heart, while cell cycle, DNA repair, mitochondrial function, oxidative phosphorylation, fatty acid metabolism, cardiac contractility and relaxation, vasculogenesis, autophagy, and proteasome pathways were downregulated (Tables S5 and S6). Notably, the generalized increase in chronic inflammatory processes of the innate immune system along with downregulation of pathways relevant to cardiac muscle mechanics and vascular growth highlights the parallels between cardiac aging biology and leading HFpEF hypotheses, which have proposed a central role for microvascular inflammation inducing the cardiomyocyte dysfunction seen in HFpEF (Paulus & Tschope, 2013).

Comparative analysis between the aging (4 mo versus 30 mo) and ExT (30 mo sedentary vs. 30 mo ExT) cohorts identified 216 pathways, which were regulated in opposing directions by aging and ExT (Tables S2, S3, S5 and S6). Of these 216 pathways, the most highly significant changes predominantly occurred in cell cycle or cell division pathways (Figure 5c), suggesting that reversing impairments in this hallmark of aging (Lopez‐Otin, Blasco, Partridge, Serrano, & Kroemer, 2013) may be an important contributor by which ExT improves the performance of the aged heart. The main drivers of the three most highly upregulated pathways (cell cycle process, cell cycle, mitotic cell cycle) were *HAUS8, GADD45A, MAPRES2, MCM5,* and *FANCI*. Increased expression trends of these genes were validated by QPCR in an independent cohort of old male mice that underwent eight weeks of voluntary wheel running (Figure S6a), suggesting that different modes of aerobic ExT can induce similar biological effects in the aged heart. Although the changes were not as robust as the cell cycle changes noted above, ExT also reversed the downregulation of ubiquitin–proteasome, cellular stress response, heat shock protein binding, and fatty acid metabolism pathways associated with aging (Tables S2, S3, S5 and S6).

## 3 DISCUSSION

HFpEF is a clinical syndrome with high morbidity and mortality, most commonly seen in older adults (Dunlay et al., 2017). Given the lack of effective pharmacological therapies for this disease, along with its projected future growth with ongoing population aging, HFpEF has been labeled as one of the largest unmet needs in cardiovascular medicine (Parikh et al., 2018). The reason for the lack of effective therapies in HFpEF is multifold, but largely due to an incomplete understanding of its complex pathophysiology.

This study addresses one of the major shortcomings in this field, which is the limited number of animal models to identify and study causal molecular mechanisms in HFpEF (Roh et al., 2017). Previous studies have suggested that although aged mice exhibit some features in common with HFpEF, they do not necessarily model HFpEF (Dai et al., 2009; Eisenberg et al., 2016). The mere presence of cardiac HFpEF phenotypes in mice does not necessarily equate to the clinical syndrome of HF, which is inherently difficult to ascertain in animals. However, our comprehensive functional, histological, and molecular phenotyping provides strong evidence that the aged C57BL/6 male mouse captures many of the major cardiac phenotypes that have been implicated as core pathophysiologic mediators of HFpEF (Borlaug, 2014). Importantly, the HFpEF phenotypes observed in aged C57BL/6 male mice occur in the absence of overt hypertension, which has been a common adjunct intervention used to generate HFpEF phenotypes in animal models (Eisenberg et al., 2016; Hulsmans et al., 2018; Schiattarella et al., 2019). Thus, we propose that the aged C57BL/6 male mouse not only represents a valuable and complementary model of HFpEF but is particularly well suited for studying the role of aging biology in HFpEF pathophysiology. This supports an emerging paradigm in this field, which suggests that given the heterogeneity in HFpEF, effective therapeutic discovery and implementation will likely need to focus on specific subgroups (e.g., older adults) in which the primary drivers of HFpEF may differ (Shah et al., 2016).

The second major aim of this study was to begin to elucidate biological mechanisms by which ExT potentially alters cardiac aging phenotypes that contribute to HFpEF in older adults. Aerobic ExT and caloric restriction have been the only interventions to improve functional capacity in older HFpEF patients in randomized clinical trials (Kitzman et al., 2016). However, due to limited access to cardiac tissue, the molecular mechanisms by which these interventions induce these benefits have remained largely unknown. Here, we focused specifically on ExT with the hypothesis that it would induce similar functional benefits in the aged C57BL/6 male mouse and that RNAseq analyses of cardiac tissue would not only provide mechanistic insights into the role of ExT in cardiac aging, but also potentially identify much‐needed novel therapeutic targets for HFpEF. Indeed, our findings show that although ExT only improves some cardiac HFpEF phenotypes in this model, it improves overall cardiac performance and exercise capacity. We propose that the functional parallels seen with ExT in these mice and humans with HFpEF not only provide further support for the use of the aged C57BL/6 male mouse as a model of age‐related HFpEF, but also for the use of ExT as a platform for therapeutic discovery in this disease.

Importantly, these are the first data to assess how ExT modulates the transcriptome of the aged heart in the context of functional phenotyping. Our analyses provide two major mechanistic insights into the therapeutic role of ExT in age‐related HFpEF. First, in the aged male heart, ExT does not significantly regulate biological pathways associated with conventional HF drugs, including β‐blockers and RAAS inhibitors. This is consistent with the failure of pharmacological therapies targeting these pathways to improve HFpEF outcomes in older adults. Borderline significance toward enhanced production or cellular responses to NO was detected in our pathway analysis, which could suggest that NO biology may still be a promising therapeutic target for age‐related HFpEF. However, given the neutral results in randomized controlled trials with organic nitrate and inorganic nitrite therapies (Borlaug et al., 2018; Redfield et al., 2015), alternative approaches to targeting this biology need to explored. Second, in contrast to its lack of effect on the transcriptional profile of neurohormonal pathways, ExT induced a marked reversal in the global downregulation of cell cycle‐related pathways seen in the aged heart (Figure 5c). This study was not designed to determine which cell populations this signal pertains to. However, the increase in capillary density and upregulation of angiogenesis pathways, in the absence of cardiac mass or fibrosis changes, suggest that endothelial proliferation is likely involved, which would be consistent with previous reports on ExT‐induced cardiac angiogenesis (Lemitsu, Maeda, Jesmin, Otsuki, & Miyauchi, 2006) and the trends seen in NO pathways. It is possible that ExT‐induced cardiomyogenesis may also be contributing to this signal. Recent work from our group has shown that aerobic ExT generates a robust increase in cardiomyogenesis in young mice (Vujic et al., 2018). Although this has yet to be studied in old animals, we have found that exercise‐mediated molecular drivers of cardiomyogenesis not only modulate proliferation processes, but often promote other pro‐survival and protective growth pathways in cardiomyocytes (Bostrom et al., 2010; Liu et al., 2015). Given the profound cardiomyocyte loss and reduced regenerative capacity in the aged heart (Bergmann et al., 2009), even small increases in cardiomyogenesis would likely have substantial impacts on cardiac function.

At an individual gene level, after adjusting for multiple hypothesis testing, our RNAseq analyses did identify 11 candidates that were differentially regulated by ExT in the aged heart (Figure 5a, Table S1). Of these 11 candidates, the increases in *SORL1* and *ACTA1* expression were fully validated by QPCR in an independent ExT cohort of old mice (Figure S6b). *SORL1* encodes for the sortilin‐like receptor 1, a low‐density lipid receptor, whose downregulation has been implicated in age‐related Alzheimer disease (Rogaeva et al., 2007). Although *SORL1* has yet to be studied in the context of cardiac aging, HF, or exercise, given its role in endosomal protein recycling, it is possible that its upregulation by ExT could mitigate some of the impaired proteostasis seen in cardiac aging and HF. The ExT‐induced upregulation of cardiac *ACTA1* expression in aged mice was unexpected. *ACTA1* is a member of the “fetal" gene profile typically increased in *pathological* cardiac hypertrophy and downregulated in exercise‐induced *physiological* hypertrophy (Vega, Konhilas, Kelly, & Leinwand, 2017). However, high‐intensity ExT can increase *ACTA1* expression in the heart (Castro et al., 2013). It is plausible that even though our ExT protocol was initially graded as moderate intensity, it became progressively more strenuous for the old animals as they aged over 8 weeks. Although we did not detect a significant difference in cardiac mass in our ExT old mice, average cardiomyocyte size increased by ~1.4‐fold, which would be consistent with the increased cardiac *ACTA1* expression observed with ExT. Further work is needed to determine whether exercise intensity has differential effects on the “fetal gene” profile associated with pathologic cardiac hypertrophy. Importantly, these data also raise the question of whether the fetal gene expression profile can reliably distinguish between physiologic and pathologic hypertrophy in older animals and humans. Evidence in humans has suggested that moderate intensity distance running specifically increases circulating BNP, another member of the pathologic cardiac hypertrophy fetal gene profile, in older, but not younger humans (Kim et al., 2017). In our ExT old mice, the improvements in cardiac function and absence of fibrotic changes suggest that despite an overall upregulation in the fetal gene expression profile, exercise appears to induce a beneficial effect in the aged murine heart. Lastly, it is important to note that our RNAseq analyses did not identify significant transcriptional changes in targets that have been previously reported in ExT aged rodents, such as SERCA2a, VEGF, and SIRT1 (Lai et al., 2014; Lemitsu et al., 2006; Tate et al., 1996). However, it is likely that some of these targets, such as SERCA2a, are largely regulated at a post‐transcriptional level in the aged heart (Roh et al., 2019).

Some limitations of the study warrant emphasis. First, this study was done exclusively in male mice and, thus, does not address sex‐related differences in age‐related HFpEF. Evidence suggests that there are likely molecular differences in how male and female hearts age, and moreover, how they remodel in response to physiologic and pathologic stress (Konhilas et al., 2004; Piro, Della Bona, Abbate, Biasucci, & Crea, 2010; Weinberg et al., 1999). While our findings strongly suggest that the aged C57BL/6 male mouse recapitulates many of the clinical HFpEF phenotypes, further work needs to be done to determine whether HFpEF phenotypes are also present in aged female mice, and if cell cycle pathways are similarly modulated by age and exercise in females. Second, although we included many of the core pathophysiologic features in our HFpEF phenotyping, this did not include assessment of other potentially causal comorbidities, such as obesity, or the role of peripheral mechanisms in HFpEF pathophysiology (Borlaug, 2014; Kitzman et al., 2016). Noncardiac phenotypes likely contribute to the age‐related exercise intolerance seen in this model and need to be further investigated. Third, while no differences in systemic arterial pressure were detected between young and old mice, we did not assess for changes in aortic stiffness, which increases with age and could also be contributing to the pathologic cardiac hypertrophy phenotype seen in this aged mouse model (Fleenor et al., 2014). Lastly, altered pathways were identified using RNAseq on whole heart extracts. Thus, while this is the first ExT‐related transcriptome analyses in aged hearts, future studies will need to define the specific cell populations driving the functional and molecular changes induced by ExT in the aged heart. Moreover, RNAseq does not identify regulation that occurs at the level of protein expression or post‐translational modifications of protein.

Taken together, this study addresses some of the major shortcomings in HFpEF research, particularly in the context of aging. It establishes the aged C57BL/6 male mouse as a valuable model for studying the role of aging biology in HFpEF pathophysiology. Moreover, by using ExT as a platform for therapeutic discovery, it identifies multiple pathways implicated in cardiac aging, most notably impaired cell cycle‐related pathways, that may potentially represent promising targets for therapeutic development in age‐related HFpEF.

## 4 EXPERIMENTAL PROCEDURES

### 4.1 Mice

All animal studies were approved by the Beth Israel Deaconess Medical Center and Massachusetts General Hospital Institutional Animal Care and Use Committees. Aged C57BL/6 males were generously provided by the National Institute on Aging. Aged C57BL/6 females were unavailable at the time of this study. Young C57BL/6 males were purchased from Jackson Laboratory.

### 4.2 Echocardiography

Echocardiography was performed on unanesthetized mice with Vivid 7 and E90 systems (GE Healthcare). Systolic function was assessed by fractional shortening and radial systolic strain, while diastolic function was assessed by early diastolic strain rate. Refer to supplemental methods for details on echocardiographic image acquisition and analysis.

### 4.3 Cardiac magnetic resonance imaging

Mice were anesthetized with isoflurane and imaged using a 9.8‐T MRI system (Bruker Biospin). Refer to supplemental methods for details of the cardiac MRI protocol.

### 4.4 Invasive intracardiac hemodynamics

Mice were anesthetized with isoflurane and mechanically ventilated throughout the procedure. The LV was entered via the right carotid artery with a Scisence 1.2F high‐fidelity micromanometer catheter (Transonic Systems Inc.) to record pressure‐volume (PV) loops. PV loops were analyzed off‐line with LabScribe2 software (iWorx).

### 4.5 Stress echocardiography exercise testing

To measure exercise capacity and cardiac reserves, a stress echocardiography protocol was designed in which mice were run to exhaustion and then immediately imaged via echocardiography. Refer to the supplemental methods for details of the protocol.

### 4.6 Exercise training protocols

Two aerobic exercise training protocols were used in this study. In the initial discovery cohort, moderate‐intensity treadmill running was performed five days per week for eight consecutive weeks. Treadmill running was done on an automated treadmill (Columbus Instruments) at a constant speed of 10 m/min at 10° incline. To ensure that the biological effects of ExT were not limited to a specific type of aerobic exercise, we also performed eight consecutive weeks of voluntary wheel running (STARR Life Sciences) in an independent validation cohort, using previously published methods (Vujic et al., 2018).

### 4.7 Histologic and immunohistochemical analyses

Formalin‐fixed, paraffin‐embedded mid‐ventricular sections were stained with periodic acid–Schiff for cardiomyocyte cross‐sectional area (CSA), Masson's trichrome for fibrosis, and rabbit‐anti‐mouse CD31 (1:50, Cell Signaling Technologies, #77699) for capillary density. Cardiomyocyte CSAs were measured in three to five random sections (40–60 cells/section, ~200 cells/heart), which were averaged to represent a single data point for each heart. Capillary density was quantified by dividing the number of CD31+ cells by the area of randomly selected sections. Three to five sections were measured per heart and averaged to represent a single data point. Given the extensive variability in fibrosis distribution throughout the heart, BZ‐X Analyzer software (Keyence) was used to quantify fibrosis in full mid‐ventricular sections. Percent fibrosis was calculated as the ratio of fibrotic area to total tissue area. Measurements from two sections were averaged to represent a single data point for each heart. Quantitative histologic analyses were done in a blinded fashion.

### 4.8 Quantitative real‐time PCR

Real‐time PCR products were carried out using SYBR‐green and standard amplification protocols. Expression levels were calculated using the ΔΔCt method. Primer sequences are listed in Table S7.

### 4.9 RNA sequencing

RNA sequencing was performed by the MGH Sequencing Core. Libraries were constructed from polyA‐selected RNA using a NEBNext Ultra Directional RNA Library Prep Kit (New England Biolabs) and sequenced on Illumina HiSeq2500 instrument. The R package DESeq2 was used for differential gene expression analysis. Genes were considered differentially expressed if upregulated by log_2_FC>+1 or downregulated by log_2_FC<−1 with an adjusted *p* value < .05 (using Benjamini‐Hochberg correction). Pathway analysis was performed with Gene Set Enrichment Analysis (GSEA, Broad Institute) using the Gene Ontology database. For all differentially expressed genes, a metric was computed as the product of logFC and −log_10_(*p*‐value). A “running sum” statistic was calculated for each gene set in the pathway database based on the ranks of the members of the set relative to those of the nonmembers. Enrichment score (ES) was defined as the maximum sum of the running sum with the genes making up this maximum ES contributing to the core enrichment in that pathway. A normalized enrichment score (NES) was generated based on the gene set enrichment scores for all dataset permutations. Pathways with a false discovery rate (FDR) <0.25 were considered significant.

### 4.10 Statistical analyses

RNAseq and GSEA data analyses were performed with R and DESeq2 software, as described in Section 4.9. GraphPad Prism (version 7.0) was used for all other data analyses. In all graphs, data are shown as means ± *SEM* with all individual data points displayed. For comparisons of two groups, unpaired Student's *t* tests were performed. For comparisons of ≥3 groups, one‐way ANOVA followed by post hoc Tukey's multiple comparison testing was done. Pearson method was used for correlation studies in Figure 2. *p* value <.05 was considered statistically significant.

## CONFLICT OF INTEREST

None declared.

## AUTHORS’ CONTRIBUTIONS

JDR, NH, and AR designed the study. JDR, NH, AYu, HL, CL, AV, FD, DZ, RS, and MJH performed the in vivo experiments. JDR, NH, AYu, BC, AYe, HL, RH, VC, CL, AV, CP, FD, DZ, RS, MJH, and RTL assisted with tissue analyses and/or data interpretation. JDR and AR wrote the manuscript with contributions from all authors.

## Supporting information

Supplementary Material

Table S1‐S7

## Data Availability

The data that support the findings of this study are all present in the paper or the Supplemental Materials. The raw RNA sequencing data used in this study will be available in the NCBI SRA repository.
