# Supplementary material for: Exercise training reverses cardiac aging phenotypes associated with heart failure with preserved ejection fraction in male mice
Source: Aging Cell. 2020 May 22;19(6):e13159. doi: 10.1111/acel.13159 (PMC7294786; doi:10.1111/acel.13159)
Supplement: Supplementary file 1 — Supplementary Material [file ACEL-19-e13159-s001.pdf]

SUPPLEMENTAL MATERIALS:

SUPPLEMENTAL METHODS:

Cardiac Magnetic Resonance Imaging (MRI)

Mice were imaged using a 9.8-T MRI system (Bruker Biospin) with a phased array rat head coil. Mice were anesthetized with isoflurane (induction, 4%–5%; maintenance, 1%–2.5%) and positioned prone on a water-heated bed in the MRI bore. Cardiac MRI images were acquired with ECG and respiratory gating (Model 1025 Monitoring & Gating System, Small Animal Systems, Inc). LV size and function were assessed using short-axis cine gradient-echo imaging with full LV coverage (repetition time TR 5.9 msec; echo time TE 2.2 msec; 12-15 msec per cardiac phase; in-plane spatial resolution of 100-120 μm × 180-210 μm; 1 mm slice thickness, no gap). Epicardial and endocardial contours at end-systole and end-diastole were traced with dedicated cardiac MRI software (MASS research CMR software, Leiden University Medical Center), to quantitate the LV end-diastolic volume (LVEDV), LV end-systolic volume (LVESV), and LV mass using Simpson rule. LV ejection fraction (EF) was calculated as [(LVEDV-LVESV)/LVEDV] x 100.

Echocardiography

Transthoracic echocardiography was performed using either a Vivid7 or E90 system equipped with a high frequency linear array transducer (i13L or L8-18i-D) (GE Healthcare). Given the known depressive effects of anesthesia on cardiac inotropic and chronotropic function, all echocardiography was performed in non-sedated mice. Mice were acclimated to the procedure at least three independent times before formal data collection. Echocardiographic images were acquired with the mouse held in a prone position with the transducer facing upward onto the chest wall.

Parasternal short-axis M-mode images at the papillary muscle level were acquired at a depth of 10 mm to measure mid LV dimensions at end-diastole (LVEDD) and end-systole (LVESD). Anterior (AWT) and posterior wall thickness (PWT) dimensions were measured at end-diastole. Heart rate was averaged from three consecutive beats from three independent images (total nine beats).

Parasternal short-axis 2D images were acquired at ~400-600 frames per second for Speckle tracking strain analysis. Radial strain analysis was performed offline using GE EchoPACS software (version 201) on optimal 2D cine images in which endocardial borders could be well delineated in three consecutive cardiac cycles. Using the software's semi-automated tracing system, the region of interest was generated by tracing the endocardial and epicardial borders at end-diastole. LV radial strain and strain rate curves were then generated for six segments at the papillary muscle level for a full cardiac cycle set from end-diastole to end-diastole. Systolic strain was averaged from the peak value of segmental strain curves in systole. Early diastolic strain rate was averaged from the six segmental strain rates at 33% into diastole. Diastole was defined from aortic valve closure (AVC) to maximum LV dimension at end-diastole, and segmental early diastolic strain rates were then measured from the frame corresponding to 33% between AVC and end-diastole and then averaged.

Cardiac systolic function was assessed by fractional shortening (FS=[(LVEDD-LVESD)/LVEDD] x 100) and radial systolic strain. Echocardiographic assessment of diastolic function was done by early diastolic strain rate, which measures the rate of myocardial deformation during the active myocardial relaxation phase. This method was selected because it is less angle- and load-dependent than other methods, and enabled us to assess diastolic function in animals without sedation. A limitation of this study is that conventional diastolic function techniques, such as mitral inflow (E/A) ratios and E/e' ratios, could not be obtained due to physiological heart rates in the 500-700 bpm range and inability to acquire apical views without the use of anesthesia.

Stress Echocardiography Protocol

A modified stress echocardiography protocol was designed to measure exercise capacity and cardiac reserves in mice. The protocol was completed over two days with day 1 dedicated to resting baseline echocardiography and day 2 dedicated to exercise capacity testing and echocardiographic assessments at peak exercise.

Baseline Echocardiography:

Baseline resting echocardiography, as described in the echocardiography methods section above, was performed on a separate day to avoid any potential confounding effects on exercise testing.

Exercise capacity testing:

Prior to formal testing, mice were acclimated to the automated treadmill (Columbus Instruments). Animals were acclimated for three consecutive days by walking at a pace of 5-10 m/min for 5 min (day 1), 10 min (day 2), and 15 min (day 3). The treadmill protocol for exercise capacity consisted of a warmup phase and a run-to-exhaustion phase with the treadmill incline set at 10°. Warmup lasted for 5 min, during which the treadmill speed increased from 5 m/min to 15 m/min. The run phase started at a speed of 15 m/min and increased at a pace of 2 m/min/min until exhaustion was reached. Animals were motivated to run with a combination of tail tapping and puffs of compressed air directed at the hindlimbs. An animal was judged to be exhausted if it could not keep pace with the treadmill for a full three seconds without falling back on to the resting pad, a pattern that had to be repeated three times in a row. Once exhaustion was reached, a small amount of blood was immediately acquired by nicking the tip of the tail with a sharp razor blade. Maximum exercise effort was then corroborated by measuring point-of-care blood lactate with a StatStrip Xpress Lactate Meter (Nova Biomedical).

Echocardiography at Peak Exercise

Immediately after peak lactate measurement, animals were rapidly transferred to an echocardiographer who acquired parasternal short-axis M-mode images within 30 seconds of peak exercise. Chronotropic reserves were assessed by measuring HR at peak exercise, which was an average of nine beats (three consecutive beats from three independent images). Contractile reserves were assessed by comparing FS at peak exercise to baseline FS at rest (from day 1) to determine the degree of FS augmentation in response to exercise

Blood Pressure Measurement:
Systemic blood pressure was measured using a tail cuff approach with the CODA noninvasive blood pressure system (Kent Scientific). Mice were acclimated to the system and final measurements were done without the use of anesthesia. At least nine blood pressure measurements were obtained for each mouse, and then averaged, to provide a single averaged blood pressure per mouse.

SUPPLEMENTAL FIGURES:

Supplemental Figure 1:

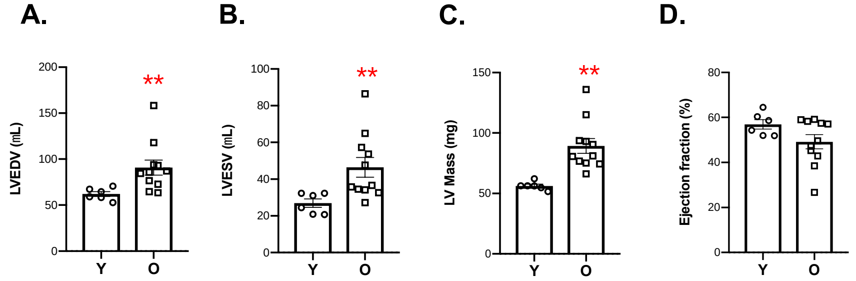

Supplemental Figure S1: Cardiac structure and function phenotyping with cardiac magnetic resonance (CMR) imaging. CMR, the gold standard imaging study for cardiac volume and ejection fraction assessments, was performed in a subgroup of young (Y, 3-4mo, n=6) and old (O, 24-26mo, n=11) C57BL/6 male mice. A) Left ventricular end-diastolic volume (LVEDV). B) Left ventricular end-systolic volume (LVESV). C) Left ventricular mass calculated using the Simpson's rule. D) Left ventricular ejection fraction. Data shown as mean ± SEM, with inclusion of all individual data points. Unpaired Student's t test used for analyses. * p<0.05, ** p<0.01, *** p<0.001.

Supplemental Figure 2

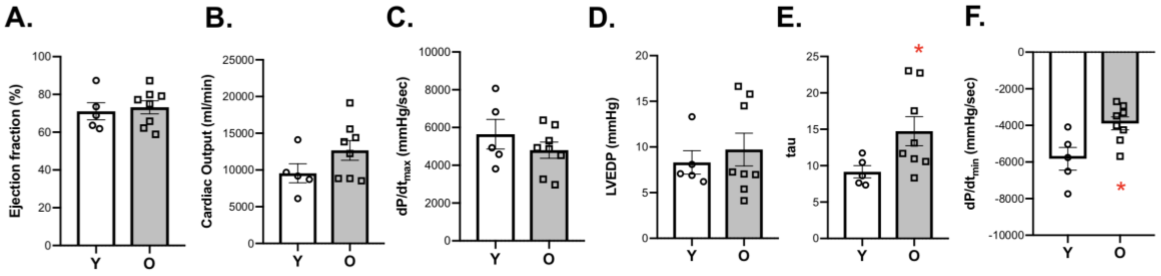

Supplemental Figure S2: Cardiac function phenotyping with invasive intracardiac hemodynamics. Invasive intracardiac hemodynamic testing, the gold standard for murine cardiac function assessments, was performed in a subgroup of young (Y, 3-4mo, n=5) and old (O, 24-26mo, n=8) C57BL/6 male mice to validate the echocardiography-based cardiac phenotyping. Cardiac systolic function was measured by A) Left ventricular ejection fraction, B) Cardiac output, and C) Maximum dP/dt. Cardiac diastolic function was measured by C) Left ventricular end-diastolic pressure (LVEDP), D) the relaxation constant, *tau*, and E) Minimum dP/dt. Data shown as mean ± SEM, with inclusion of all individual data points. Unpaired Student's t test used for analyses. * p<0.05, ** p<0.01, *** p<0.001.

Supplemental Figure 3:

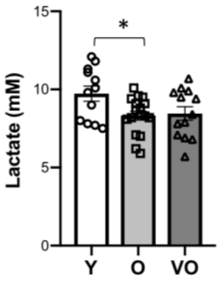

Supplemental Figure S3: Peak lactate measurements during exercise testing. Baseline stress-echocardiography testing in C57BL/6 male mice at 3-4 months (young (Y), n= 12), 24-26 months (old (O), n=17), and 28-30 (very old (VO), n=13). Peripheral blood lactate measurements were obtained at peak exercise to confirm exhaustion and adequate effort during exercise testing. Data shown as mean ± SEM, with all individual data points plotted. One-way ANOVA with post-hoc Tukey's test used for analyses. * p<0.05, ** p<0.01, *** p<0.001.

Supplemental Figure 4:

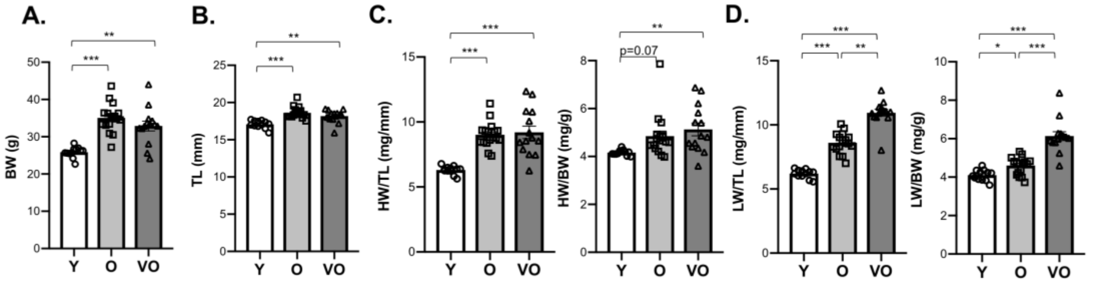

Supplemental Figure S4: Gravimetric analysis of C57BL/6 mice from ages 4 to 30 months. Gravimetric measurements in 3-4 month (young (Y), n=12), 24-26 month (old (O), n=17), and 28-30 month (very old (VO), n=14). A) Body weight (BW). B) Tibial length (TL). C) Heart weight (HW) normalized to TL or BW. D) Lung weight (LW) normalized to TL or BW). Data shown as mean ± SEM, with all individual data points plotted. One-way ANOVA with post-hoc Tukey's test used for analyses. * p<0.05, ** p<0.01, *** p<0.001.

Supplemental Figure 5:

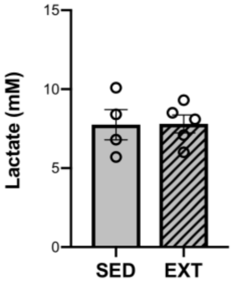

Supplemental Figure S5: Peak lactate measurements during exercise testing in ExT substudy. Final stress echocardiography testing was performed on 4 sedentary and 5 exercise trained (ExT) mice due to deaths prior to study completion. All animals were 30 months old at study completion. Peripheral blood lactate measurements were obtained

at peak exercise to confirm exhaustion and adequate effort during exercise testing. Data shown as mean ± SEM, with all individual data points plotted. Unpaired Student's t-test used for analyses. * $p<0.05$, ** $p<0.01$, *** $p<0.001$.

Supplemental Figure S6

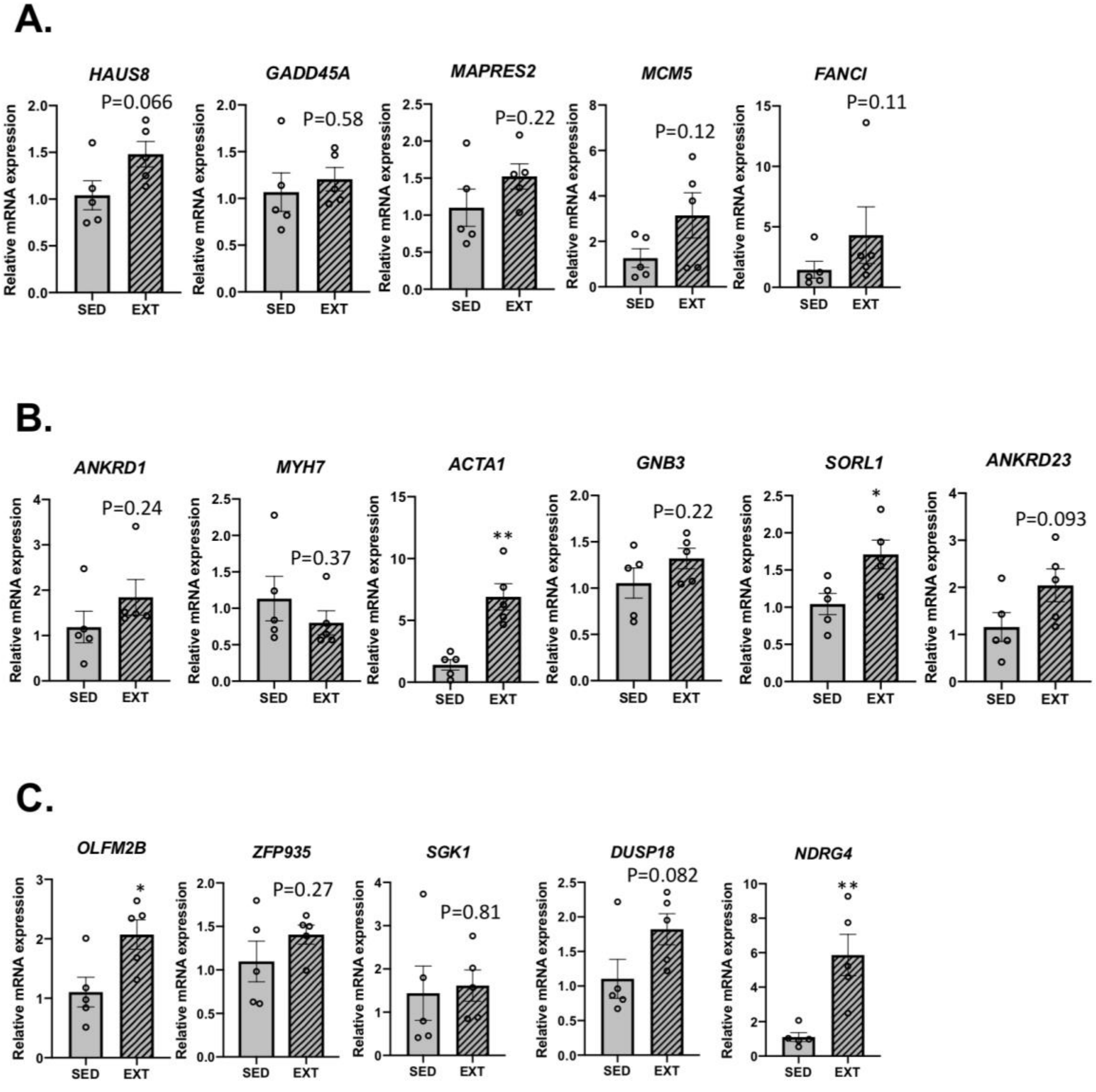

Supplemental Figure S6: QPCR validation of ExT RNAseq candidates. Validation was done in cardiac samples from an independent cohort of 18-month-old C57BL/6 males subjected to eight weeks of voluntary wheel running (EXT) versus normal sedentary lifestyle (SED). n=5/group. A) Top five genes driving cell cycle-related pathway enrichment. B) Genes significantly upregulated by ExT in discovery cohort. C) Genes significantly downregulated by ExT in discovery cohort. Data shown as mean ± SEM, with all individual data points plotted. Unpaired Student's t-test used for analyses. * $p<0.05$, ** $p<0.01$, *** $p<0.001$.

SUPPLEMENTAL TABLE LEGENDS:

Please refer to separate .xlxs file “ACE-19-0609 Revision_Supplemental Tables S1-S7” for supplementary tables.

Supplemental Table S1: Cardiac transcriptome profile in sedentary versus exercise-trained aged mice. Comparison of RNAseq profiles of cardiac tissue from very old (30 month) sedentary versus very old (30 month) exercise trained (ExT) mice. n=3 per group. ExT mice completed eight weeks of moderate-intensity daily treadmill running from 28 to 30 months, prior to tissue collection. Multiple hypothesis testing done with Benjamini Hochberg method. $P_{adj} < 0.05$ considered significant.

Supplemental Table S2: Pathways upregulated in aged hearts with exercise training. Gene set enrichment analysis performed on cardiac RNAseq profiles from very old (30 month) sedentary versus very old (30 month) exercise-trained mice using the Gene Ontology pathway database. n=3/group. For each pathway, data presented as enrichment score (ES) followed by false discovery rate (FDR) value. FDR value < 0.25 considered significant. NA = not applicable (for pathways downregulated).

Supplemental Table S3: Pathways downregulated in aged hearts with exercise training. Gene set enrichment analysis performed on cardiac RNAseq profiles from very old (30 month) sedentary versus very old (30 month) exercise-trained mice using the Gene Ontology pathway database. n=3/group. For each pathway, data presented as enrichment score (ES) followed by FDR value. FDR value < 0.25 considered significant. NA = not applicable (for pathways upregulated).

Supplemental Table S4: Cardiac transcriptome profile in young versus very old mice. Comparison of cardiac RNAseq profiles of young (4 month) versus very old (30 month) sedentary mice. n=3 per group. Multiple hypothesis testing done with Benjamini Hochberg method. $P_{adj} < 0.05$ considered significant.

Supplemental Table S5: Pathways upregulated in cardiac tissue with age. Gene set enrichment analysis performed on cardiac RNAseq profiles from young (4 month) versus very old (30 month) sedentary mice using the Gene Ontology pathway database. n=3/group. For each pathway, data presented as enrichment score (ES) followed by FDR value. FDR value < 0.25 considered significant. NA = not applicable (for pathways downregulated).

Supplemental Table S6: Pathways downregulated in cardiac tissue with age. Gene set enrichment analysis performed on cardiac RNAseq profiles from young (4 month) versus very old (30 month) sedentary mice using the Gene Ontology pathway database. n=3/group. For each pathway, data presented as enrichment score (ES) followed by FDR value. FDR value < 0.25 considered significant. NA = not applicable (for pathways upregulated).

Supplemental Table S7: Primer sequences used for QPCR.
